# Expression of GLUT1 and GLUT3 Glucose Transporters in Endometrial and Breast Cancers

**DOI:** 10.1007/s12253-012-9500-5

**Published:** 2012-01-21

**Authors:** Anna Krzeslak, Katarzyna Wojcik-Krowiranda, Ewa Forma, Paweł Jozwiak, Hanna Romanowicz, Andrzej Bienkiewicz, Magdalena Brys

**Affiliations:** 1 Department of Cytobiochemistry, University of Lodz, Pomorska 141/143, 90-236 Lodz, Poland; 2 Department of Gynecological Oncology, Medical University of Lodz, Pabianicka 62, 93-509 Lodz, Poland; 3 Department of Clinical Pathomorphology, Polish Mother’s Memorial Hospital Research Institute, Łodz, Rzgowska 281/289, 93-338 Łodz, Poland

**Keywords:** Glucose transporters, mRNA expression, Protein expression, Breast cancer, Endometrial cancer

## Abstract

Cancer cells have accelerated metabolism and high glucose requirements. The up-regulation of specific glucose transporters may represent a key mechanism by which malignant cells may achieve increased glucose uptake to support the high rate of glycolysis. In present study we analyzed the mRNA and protein expression of GLUT1 and GLUT3 glucose transporters by quantitative real-time polymerase chain reaction (Q-PCR) and Western blotting technique in 76 cases of endometrial carcinoma and 70 cases of breast carcinoma. *SLC2A1* and *SLCA2A3* mRNAs expression was found, respectively in 100% and 97.4% samples of endometrial cancers and only in 50% and 40% samples of breast cancers. In endometrial cancers GLUT1 and GLUT3 protein expression was identified in 67.1% and 30.3% of cases. Analogously, in breast cancers in 48.7% and 21% of samples, respectively. The results showed that both endometrial and breast poorly differentiated tumors (grade 2 and 3) had significantly higher GLUT1 and GLUT3 expression than well-differentiated tumors (grade 1). Statistically significant association was found between *SLCA2A3* mRNA expression and estrogen and progesterone receptors status in breast cancers. GLUT1 has been reported to be involved in the uptake of glucose by endometrial and breast carcinoma cells earlier and the present study determined that GLUT3 expression is also involved. GLUT1 and GLUT3 seem to be important markers in endometrial and breast tumors differentiation.

## Introduction

Glucose uptake and glycolytic metabolism are enhanced in cancer cells compared to normal cells [1, 2]. This observation was first reported by Otto Warburg several decades ago [3, 4]. He hypothesized that conversion of glucose into lactate combined with suppression of mitochondrial function reflect the major metabolic change in malignant transformation [3, 4].

Transport of glucose across the plasma membrane is the first rate-limiting step for glucose metabolism and is mediated by facilitative glucose transporter proteins (GLUTs) [5, 6]. Fourteen members of the glucose transporter family have been identified. The genes belong to the solute carrier 2A family with gene symbol *SLC2A*. The glucose transporters differ in their tissue distribution and each transporter protein possesses different affinities for glucose and other hexoses such as fructose [5, 6]. GLUT1 is broadly expressed in the body tissues and is involved in glucose uptake in the basic state [6]. Elevated level of GLUT1 have been shown in almost all human cancers including brain, breast, head and neck, bladder, renal, colorectal, lung and ovarian cancers [6]. Some studies have demonstrated that the level of GLUT1 expression correlates with specific tumor characteristics. Comparatively higher levels of expression are seen in cancers of higher grade and proliferative index and in cancers of lower degree of differentiation. GLUT1 expression has been associated with increased malignant potential, invasiveness and poor prognosis in lung, colorectal, gastric and ovarian cancers [7–11].

GLUT3 is also expressed in human malignant tissue, but not so frequently as GLUT1. GLUT3 isoform seems to be a predominant glucose transporter in highly malignant glial cells of human brain [12]. Oligonucleotide microarray analysis revealed that *SLC2A3* (gene encoded GLUT3) overexpression was correlated with tumor size, pathologic stage and recurrence in oral tongue carcinoma [13]. GLUT3 protein expression evaluated by immunohistochemistry is indicator of poor prognosis outcome in non-small lung carcinoma, oral squamous cell carcinoma and laryngeal carcinoma [7, 14, 15]. However, GLUT3 mRNA or protein expressions are not correlated with any clinicopathological parameters in case of thyroid and ovarian cancers [16–18].

In present study we analyzed the mRNA and protein expression levels of GLUT1 and GLUT3 in endometrial and breast cancers and the relationship between their expression and clinicopathological parameters.

## Materials and Methods

### Patients and Samples

The studied materials were obtained from Department of Gynecological Oncology Copernicus Memorial Hospital, Łódź, Poland and from Department of Clinical Pathomorphology Polish Mother’s Memorial Hospital, Research Institute, Łódź, Poland.

The materials comprised samples of 76 endometrial carcinomas and 70 breast ductal carcinomas. Information regarding the clinical and pathological characteristics of the patient populations was obtained from the medical records. The endometrial and breast cancer patients characteristics are present in Tables 1 and 2, respectively. Endometrial normal tissue samples were obtained from 27 patients who had undergone hysterectomy. In the case of normal breast tissue, the material used for the study came from 36 women after a total mastectomy. All the clinical material were excised by a surgical pathologist.

**Table 1:** Characteristics of patients and endometrial cancer samples

Characteristic	Number of patients (*n* = 76)
Median age (range)	62.5 (31 – 85)
FIGO stage	
I	35
II	31
III	9
IV	1
Histological grade	
G1	14
G2	49
G3	13
Depth of myometrial invasion	
<1/2	41
>1/2	35
Hyperplasia	
No	62
Yes	14
Myomas	
No	60
Yes	16
Lymph node metastasis	
No	60
Yes	16

**Table 2:** Characteristics of patients and breast cancer samples

Characteristic	Number of patients (*n* = 70)
Median age (range)	57.3 (39–72)
Tumor grade according to Bloom-Richardson system	
I	21
II	30
III	19
Tumor size	
T1	22
T2	33
T3-T4	15
Lymph node metastasis	
No	44
Yes	26
Menopausal status	
Premenopausal	23
Postmenopausal	47
ER and PR status	
ER + PR+	33
ER-PR+/ER + PR-	16
ER-PR-	21

Endometrial carcinomas were classified according to the criteria of the International Federation of Gynecology and Obstetrics (FIGO). Histological typing and grading were done according to the WHO classification [19]. All breast carcinomas were classified according the Bloom and Richardson grading system and according TNM staging system.

### RNA Isolation and cDNA Synthesis

The tissue specimens collected in the operation room were prepared and evaluated by an experienced pathologist. Samples were stored at -80°C until RNA preparation. Total RNA was isolated using Trizol^®^ Reagent (Sigma Aldrich, USA) according to manufacturer’s protocol and quantified spectrophotometrically. First-strand cDNAs were obtained by reverse transcription of 1 μg of total RNA using RevertAid^TM^ First strand cDNA synthesis kit (Fermentas International, Lithuania) following the manufacturer’s protocol.

### Quantitative Real-Time RT-PCR

Real-time gene expression analysis of target genes (*SLC2A1* and *SLC2A3*) was performed using TaqMan^®^ Gene Expression Assays (Applied Biosystems, USA) according to manufacturer’s instructions. The *GAPDH* gene was used as internal control. The assay numbers for these genes were as follows: Hs00892681_m1, Hs00359840_m1, Hs99999905_m1.

Each PCR reaction was performed in a 10 μl volume that included 5 μl of 2x TaqMan Universal PCR MasterMix (Applied Biosystems, USA), 4.5 μl of water diluted cDNA template (50 ng) and 0.5 μl of TaqMan^®^ Gene Expression Assay consisted of a pair of unlabeled PCR primers and TaqMan probe with a FAM^TM^. The RT-qPCR reaction was carried out using the Mastercycler ep realplex (Eppendorf) under the following conditions: denaturation for 10 min at 95°C followed by 50 cycles of 15 s at 95°C, 1 min annealing and extension at 60°C.

Relative RNA quantification was performed using the ΔCt method. ΔCt (Ctgene—CtGAPDH) values were recalculated into relative copy number values (number of *SLC2A1* and *SLC2A3* mRNA copies per 1000 copies of *GAPDH* mRNA).

### *SLC2A1* and *SLC2A3* Gene Copy Number Quantification

To determine the *SLC2A1* and *SLC2A3* genes amplification, copy number quantification was carried out using quantitative real-time PCR Mastercycler ep realplex (Eppendorf) with the glucokinase (*GCK*) gene used as the reference gene. The real-time PCR primers are listed in Table 3. Real-time PCR was performed in 50 μl reaction volumes that contained 2x Power SYBR Green PCR Master Mix (Applied Biosystems, USA) and 0.9 mM forward and reverse primers. PCRs conditions were: 5 s at 95°C followed by 40 cycles consisting of 15 s at 95°C and 30 s at 60°C. ΔCt was calculated by a Ct value of GCK taking away that of *SLC2A1* and *SLC2A3* and three or more ΔCt was defined as amplified.

**Table 3:** Primers used for *SLC2A1* and *SLCA2A3* gene copy number quantification

Gene	Primer	Sequence
*SLC2A1*	Forward	TGTGCAACCCATGAGCTAA
	Reverse	CCTGGTCTCATCTGGATTCT
*SLC2A3*	Forward	TTCGTCTCTAGCCTGCACTG
	Reverse	ACACAACTTCTCCGGGTGAC
*GCK*	Forward	CGGATGCAGAAGGAGATGGA
	Reverse	CATCTTCACACTGGCCTCTTCA

### Western Blotting Analysis

The samples (50 μg protein/lane) of homogenates were resolved by 8% SDS-PAGE and electroblotted onto Immobilon-P transfer membranes (Millipore, Bedford, Massachusetts, USA). The blots were incubated 1 h with rabbit polyclonal anti-GLUT1 (Abcam, UK) or mouse monoclonal anti-GLUT3 antibodies (Santa Cruz Biotechnology. Inc., USA) in a 1:1000 and 1:400 dilution, respectively. After being washed three times with TBST (Tris buffered saline with Tween-20), the membranes were incubated 1 h with goat anti-rabbit or anti-mouse antibodies conjugated with horseradish peroxidase (1:5000 dilution). The membranes were again washed three times with TBST and incubated with peroxidase substrate solution (3,3’-diaminobenzidine -DAB). Gel-Pro^®^ Analyzer software (Media Cybernetics Inc., USA) was used for densitometry analysis of protein bands. The integrated optical density (IOD) of the bands, in a digitized picture, was measured.

### Evaluation of ER and PR

ER and PR status was determined by immunohistochemical method as part of the routine clinical practice. Using the immunohistochemical assay, tumors were classified as positive if more than 10% of the cells showed nuclear staining for the receptor. This information was received together with the characteristics of clinical material.

### Statistical Analysis

The statistical analyses were performed using the STATISTICA version 9.0 (StatSoft, Poland). Since levels of expression in endometrial and breast cancer specimens did not show normal distribution (Kolmogorov-Smirnov test) the non-parametrical statistical tests were applied (Mann–Whitney *U* test, Spearman rank analysis). Kruskal-Wallis test with post-hoc multiple comparisons were used according to clinical data. P values come from post-hoc tests. A *p*-value < 0.05 was considered as statistically significant.

## Results

### Endometrial Carcinoma

GLUT1 mRNA expression was found in all 76 samples of endometrial cancers and positive GLUT3 mRNA expression was demonstrated in 97,4% of the cases (74/76). In the case of normal tissue, GLUT1 and GLUT3 mRNA expression was observed in 22.2% (6/27) and 48.1% (13/27) samples, respectively. Mean GLUT1 and GLUT3 gene expressions in normal and cancerous tissue are presented in Table 4. The table contains p-values for comparison of normal and neoplastic tissues. The relative GLUT3 mRNA level was much lower compared to GLUT1 mRNA level.

**Table 4:** Mean *SLC2A1* and *SLC2A3* gene and GLUT1 and GLUT3 protein expression in normal and cancerous tissues. The table contains p-values for comparison of expression in normal and neoplastic tissue

	Gene [copies of gene mRNA per 1000 copies of *GAPDH* mRNA]		Protein [Integrated Optical Density]	
	*SLC2A1*	*SLCA2A3*	GLUT1	GLUT3
Endometrium				
Normal	213.4 ± 87.6	46.2 ± 24.8	124.3 ± 65.9	53.1 ± 25.4
Cancer	356.6 ± 143.7	71.3 ± 23.1	185.3 ± 43.2	71.5 ± 35.7
	*p* > 0.05	*p* < 0.05	*p* < 0.05	*p* > 0.05
Breast				
Normal	86.2 ± 21.5	58.6 ± 18.6	94.2 ± 36.7	40.7 ± 22.7
Cancer	273.7 ± 145.2	73.3 ± 30.2	137.9 ± 32.8	50.5 ± 31.5
	*p* < 0.05	*p* > 0.05	*p* > 0.05	*p* > 0.05

Amplification of the *SLC2A1* gene was found in 4% (3/76) of endometrial cancer cases, but in none of normal tissue. In the case of *SLC2A3* gene, amplification was not observed in any cancerous and normal samples. The results are summarized in Table 5.

**Table 5:** *SLC2A1* and *SLC2A3* gene amplifications in endometrial and breast cancers

Tissue type	*SLC2A1* copy number	*SLC2A3* copy number
	Three or more copies	Three or more copies
Endometrial cancer	3/76	0/76
Endometrial normal tissue	0/27	0/27
Breast cancer	7/70	2/70
Normal breast tissue	0/36	1/36

Of the carcinoma samples, 67.1% (51/76) showed GLUT1 protein expression. GLUT3 protein expression was observed in 30.3% (23/76). In case of normal tissue GLUT1 protein was detected in 26% (7/27) and GLUT3 in 14.8% (4/27) studied samples. Mean GLUT1 and GLUT3 protein expressions in normal and cancerous tissues are present in Table 4.

**Fig. 1:**
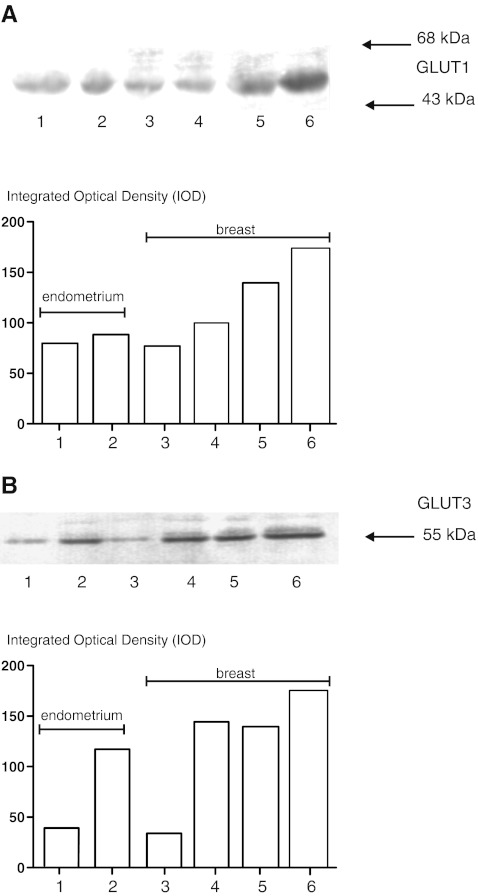
A representative results of GLUT1 **a** and GLUT3 **b** protein expression analyses in homogenates of endometrial and breast carcinomas. Lower panels show the results of quantitative densitometric analysis. **a** and **b** lane 1- normal endometrium, lane 2—endometrial carcinoma, lane 3—normal breast tissue, lanes 4–6—breast cancer samples classified according the Bloom and Richardson grading system as I, II, III, respectively

A representative results of analyses of GLUT1 and GLUT3 protein expression in homogenates of endometrial and breast carcinomas are shown in Fig. 1.

A significant correlation between the GLUT1 and GLUT3 expression levels determined by real-time PCR and those obtained by Western blotting was noted (Spearman’s rank analysis, *p* < 0.01, *p* < 0.05, respectively).

**Fig. 2:**
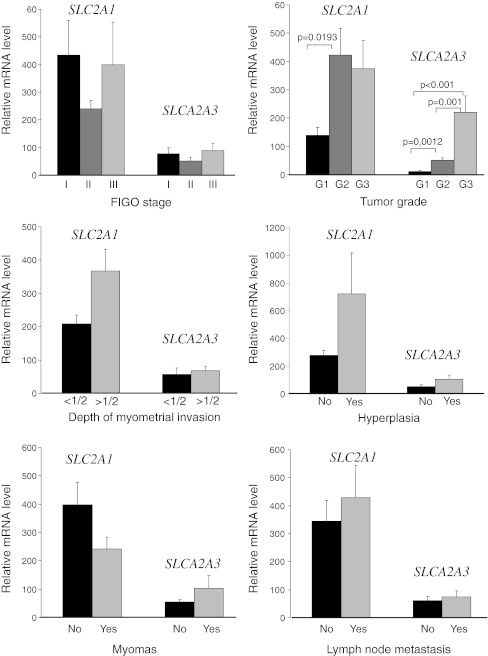
Expression of *SLC2A1* and *SLCA2A3* mRNA measured by real-time PCR in endometrial cancers *via* clinicopathological parameters. Bars indicate mean±SEM

There were no significant differences (*p* > 0.05) in GLUT1 and GLUT3 mRNA/protein expression, between tumors in different stage of development according to FIGO classification. However, the GLUT1 mRNA and protein expression was significantly higher in tumors of grade 2 than in tumors of grade 1 (*p* < 0.05). GLUT3 mRNA and protein expression increased significantly with increasing tumor grade (*p* < 0.05). There was no correlation between the other demographic and clinicopathological parameters (age, depth of myometrial invasion, hyperplasia, myomas, lymph node metastasis) and GLUT1 or GLUT3 expression. Due to the similar relationship between the GLUT1 and GLUT3 mRNAs and proteins expression levels and clinicopathological parameters, we have shown on the graphs only data for the mRNA (Fig. 2.). The values shown on the graphs apply only to cases with positive gene expression. We have also analyzed the relationship between GLUT1 and GLUT3 mRNA/protein expression, but we did not find any correlations.

### Breast Carcinoma

Positive GLUT1 and GLUT3 mRNAs expression was found respectively in 50% (35/70) and 40% (28/70) of breast cancer cases. In normal breast tissue, GLUT1 and GLUT3 mRNA expression was observed in 41.7% (15/36) and 13.9% (5/36) samples, respectively. Mean *SLC2A1* and *SLC2A3* gene expressions in normal and cancerous tissues are present in Table 4. Similarly as in the case of endometrial cancer, the relative GLUT3 mRNA level was lower compared to GLUT1 mRNA level.

The study of gene copy number showed that the *SLC2A1* gene amplification was found in 8.6% (6/70) of cancer cases, but in none normal tissue sample. In the case of *SLC2A3* gene, amplification was observed in 2.8% (2/70) of cancerous samples and in one normal tissue. The results are summarized in Table 5.

Of the carcinoma samples, 48.7% (37/76) showed GLUT1 protein expression. GLUT3 protein expression was observed in 22.8% (16/70). In case of normal tissue GLUT1 protein was detected in 22.2% (8/36) and GLUT3 in 16.7% (6/36) of studied samples. Mean GLUT1 and GLUT3 protein expressions in normal and cancerous tissue are present in Table 4. Figure 1 shows representative results of immunoblotting of GLUT1 and GLUT3 protein in breast normal and cancerous samples.

A significant correlation between the GLUT1 and GLUT3 mRNA and protein expression levels was observed (Spearman’s rank analysis, *p* < 0.05 in both cases).

**Fig. 3:**
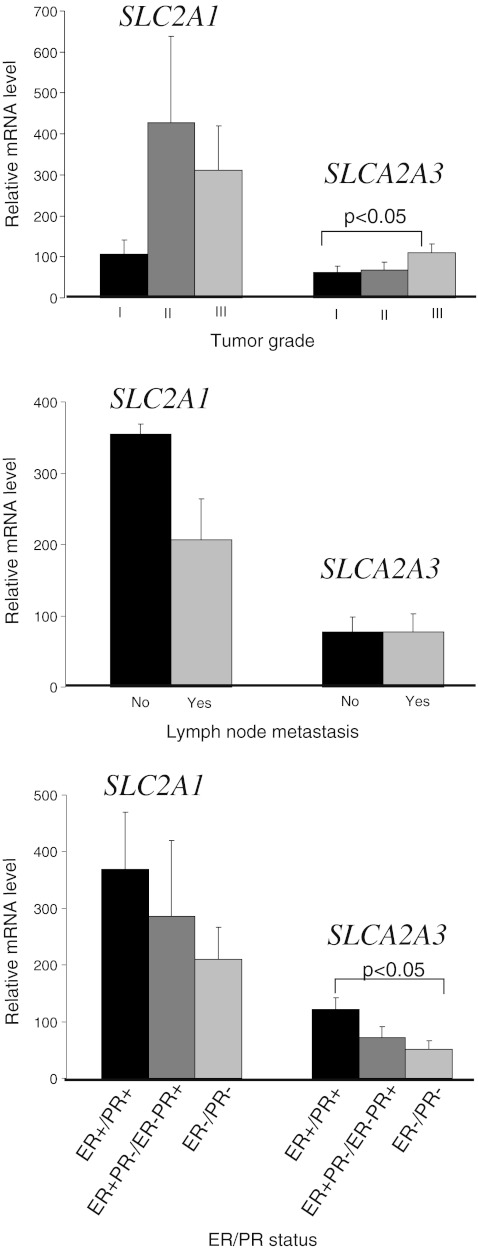
Expression of *SLC2A1* and *SLCA2A3* mRNA measured by real-time PCR in breast cancers *via* clinicopathological parameters. Bars indicate mean±SEM

There were no statistically significant differences in GLUT1 mRNA/protein expression between tumors with different demographic and clinicopathological parameters (age, tumor grade, tumoral size, lymph node metastasis, menopausal status, ER and PR status). However, the results showed a trend of poorly differentiated tumors (grade 2 and 3) to be more frequently GLUT1 positive (53,3% and 52,6%, respectively) than well differentiated one (grade 1; 38,1%). There were statistically significant differences in GLUT3 mRNA expression levels between grade 3 tumors and grade 1 tumors (*p* < 0.05). In the case of protein expression level such dependence was not showed. Statistically significant association was found between GLUT3 mRNA expression and estrogen and progesterone receptors status (Fig. 3.). For the other demographic and clinicopathological parameters correlation with GLUT3 mRNA/protein expression was not showed. Analogously as in endometrial studies, due to the similar relationship between the GLUT1 and GLUT3 genes and proteins expression levels and clinicopathological parameters, we have shown on the graphs only data for the mRNA. There was no relationship between GLUT1 and GLUT3 mRNA/protein expression in breast normal and cancerous samples.

## Discussion

Tumor cells must have increased access to glucose to support the high rate of glycolysis and satisfy their great need of energy. The up-regulation of specific glucose transporters may represent a key mechanism by which malignant cells may achieve increased glucose uptake and compensate the lack of energy caused by inefficient anaerobic glycolysis. There is little information in the literature regarding glucose transporters expression in endometrial cancers. However, the GLUT1 and GLUT8 have been reported to be involved in the uptake of glucose by endometrial carcinoma cells [20]. The results of previous immunohistochemical studies showed that GLUT1 was overexpressed in endometrial cancers compared to benign endometrial epithelium [21]. Upregulation of GLUT1 expression with increasing grade of tumors was demonstrated [20]. The results of our studies also showed higher expression of GLUT1 mRNA in less differentiated carcinomas (grade 2 and 3) compared to well-differentiated ones (grade 1).

To the best of our knowledge this study is the first documenting GLUT3 mRNA and protein expression in endometrial cancers. GLUT3 gene amplification also has not been studied so far. Relative GLUT3 mRNA expression level in endometrial carcinoma was lower than GLUT1. However, the expression of GLUT3 significantly increased with the histological grade of tumors. The expression of GLUT3 in grade 3 tumors was about 10 and 4 times higher than in grade 1 and grade 2, respectively. Our results showed no relationship between the expression of GLUT1 and GLUT3 in endometrial cancers and the number of gene copies. These results suggest that both GLUT1 and GLUT3 are involved in glucose uptake in endometrial carcinoma and they may be an important markers in tumor differentiation. There was no relationship between GLUT1 or GLUT3 mRNA expression and demographic or clinocopathological parameters such as tumor stage, coexistence of myomas and hyperplasia, lymph node metastasis. Therefore, the significance of their expression on prognosis in endometrial carcinomas requires further clarification employing a larger cohort.

Studies of GLUT1 and GLUT3 protein expression in breast cancers have given variable results. Some immunohistochemical studies detected GLUT1 expression in about 50% and the other only in 25% of breast tumors [22–24]. Younes et al. [22] demonstrated expression of GLUT1 in 42% of 118 breast tumors, with increased expression in cancers of higher grade and proliferative activity. The results of our study showed GLUT1 mRNA and protein expression in about 50% of breast cancers. There were no statistically significant differences in GLUT1 expression between tumors of different grade and stage. However, the results showed a trend of poorly differentiated tumors to be more frequently GLUT1 positive than well differentiated ones (grade 1 about 38% grade 2 and 3 about 50%). These results are similar to results of Ravazoula et al. [25] who studied 78 infiltrating ductal carcinomas and showed expression of GLUT1 in 28% of grade 1, 63.8 of grade 2 and 58.7 of grade 3 carcinomas.

Younes et al. [26] did not demonstrate GLUT3 expression in breast cancer and Godoy et al. [27] showed that GLUT3 was weakly expressed only in 3 of 33 cases of invasive ductal carcinoma and was absent in 12 normal tissues. The results of our study showed that GLUT3 mRNA expression was present in 40% of breast cancer cases. However, similarly to endometrial cancers the relative GLUT3 mRNA level was much lower than in case of GLUT1. Higher GLUT3 expression was significantly associated with poor histological grade of breast cancers. Kang et al. [23] showed that expression of GLUT1 correlated significantly with estrogen and progesterone receptors status. Our results did not show such a correlation in case of GLUT1 expression, but there was significantly higher expression of GLUT3 in estrogen and progesterone receptor positive cancers than in receptor negative carcinomas. These results are consistent with hypothesis of hormonal regulation of glucose transporters expression in cancers. Rivenzon-Segal et al. [28] found that estrogen-induced changes in glycolysis in orthotopic MCF7 human breast cancer xenografts appear to be mediated by regulation of GLUT1 expression. Increased GLUT12 protein levels after estrogen treatment in MCF-7 cells had also been demonstrated [6]. It is possible that estrogen or progesterone may influence GLUT3 expression in breast cancer as well. Amplification of the studied by us genes was found in a small number of cases and therefore we conclude that it did not affect the expression levels.

In conclusion, the expression of GLUT1 has been reported to be involved in the uptake of glucose by endometrial and breast carcinoma cells and the present study determined that GLUT3 expression is also involved. GLUT1 and GLUT3 may be important markers in endometrial and breast tumors differentiation. Clarification of the role in glucose metabolism of GLUT specific isoforms could potentially improve tumors detection and treatment.
